# Anti-PGL-I seropositivity and development of leprosy in contacts: a comprehensive analysis of sociodemographic determinants, genetic susceptibility, and exposure characteristics to *Mycobacterium leprae*

**DOI:** 10.1590/0074-02760240061

**Published:** 2025-05-26

**Authors:** Eyleen Nabyla Alvarenga Niitsuma, Isabela de Caux Bueno, Gabriel da Rocha Fernandes, Mery Natali Silva Abreu, Francisco Carlos Félix Lana

**Affiliations:** 1 Instituto Federal de Educação, Ciência e Tecnologia do Norte de Minas Gerais, Almenara, MG, Brasil; 2 Universidade Federal de Minas Gerais, Escola de Enfermagem, Belo Horizonte, MG, Brasil; 3 Fundação Oswaldo Cruz-Fiocruz, Instituto René Rachou, Grupo de Informática de Biossistemas e Genômica, Belo Horizonte, MG, Brasil; 4 Universidade Federal de Minas Gerais, Escola de Enfermagem, Departamento de Gestão em Saúde, Belo Horizonte, MG, Brasil; 5 Universidade Federal de Minas Gerais, Escola de Enfermagem, Departamento de Enfermagem Materno-Infantil e Saúde Pública, Belo Horizonte, MG, Brasil

**Keywords:** leprosy, household contacts, anti-PGL-I serology, social determinants of health, genetic susceptibility, epidemiological surveillance

## Abstract

**BACKGROUND:**

Leprosy is an infectious disease that remains hyperendemic in several Brazilian regions. Patient contacts face a higher risk for infection and illness, which can subsequently contribute to the persistence of the disease.

**OBJECTIVE:**

This study investigates the risk factors associated with anti-phenolic glycolipid-I (anti-PGL-I) seropositivity and leprosy development among contacts of leprosy patients in a highly endemic region.

**METHODS:**

A cohort of 629 contacts from the Almenara microregion, Minas Gerais, Brazil, was followed from 1998 to 2018. Our research group assessed risk factors, including sociodemographic determinants, bacillus exposure, and genetic susceptibility.

**FINDINGS:**

Analysis revealed that living with a multibacillary (MB) leprosy patient [odds ratio (OR): 3.01, 95% confidence interval (CI): 1.02-8.86] and with a patient with grade II disabilities (OR: 4.43, 95% CI: 1.08-18.1) significantly increased the likelihood of anti-PGL-I seropositivity among asymptomatic contacts. Risk factors for leprosy included living with a patient in a shared residence (OR: 2.84, 95% CI: 1.21-6.67) and blood relation to the patient (OR: 2.56, 95% CI: 1.18-5.54). Notably, 98% of contacts who developed leprosy had lived with more than one patient.

**MAIN CONCLUSIONS:**

Clinical characteristics of index patients play a critical role in infection risk among contacts. Leprosy progression appears to depend on genetic susceptibility, type of contact, and extent of bacillus exposure.

Despite substantial progress in reducing leprosy’s global burden, it remains a chronic, debilitating health issue impacting many individuals worldwide. While several countries have achieved elimination as a public health concern - defined as a registered prevalence below one case per population of 10,000 inhabitants - leprosy continues to challenge health systems in numerous regions. In response, the World Health Organization (WHO) launched the Global Leprosy Strategy 2021-2030, promoting scaled-up prevention efforts, integrated active case detection with contact tracing, and adaptable approaches tailored to different contexts and endemicity levels.^1^

In 2023, there were 174,087 newly diagnosed leprosy cases worldwide, reflecting a 6% reduction compared to 2019. However, the coronavirus disease 19 (COVID-19) pandemic disrupted leprosy control strategies in Brazil, primarily due to social distancing; interrupted surveillance activities; and suspended active case findings, thus leading to a decline in detection rates, especially among the general population and children under 15. This disruption also coincided with an increase in the detection of multibacillary (MB) cases. Following the challenges of 2020 and 2021, some priority countries, including Brazil, began to show signs of recovery, with detection rates rising in 2022 as national leprosy control programs adapted to pandemic-related obstacles.^2^ Globally, a 23.8% increase in new leprosy cases was recorded in 2023 as compared to 2021, with Brazil among the three priority countries experiencing a significant rise of 7.2% in new cases, including those among children - an indicator of recent transmission.^3^

Persistent cases and the rise in severe physical disabilities due to delayed diagnosis underscore the ongoing need for rigorous leprosy control measures. In Brazil, the epidemiological landscape of leprosy is highly variable, with subnational elimination goals yet to be uniformly achieved. Moreover, stigma toward affected individuals and the limitations of existing control strategies continue to hinder progress.^4^

Effective leprosy control relies on early detection and a thorough examination of household contacts, particularly in areas with high endemicity. While contact with leprosy patients is a significant risk factor for the disease’s persistence, exposure to *Mycobacterium leprae* alone does not fully account for infection and disease development.^5^ ^,^ ^6^ Prolonged exposure may lead to subclinical infections that heighten the risk of the onset of leprosy, thereby perpetuating the disease in endemic regions.^7^ Anti-phenolic glycolipid-I (anti-PGL-I) serology has become a valuable biomarker for detecting infection in asymptomatic contacts. Additionally, new contact-tracing strategies now incorporate advanced methods, such as electroneuromyography to assess neural damage and the detection of bacillus DNA in dermal scrapings and skin biopsies.^8^

Prior studies on contacts of leprosy patients have identified multiple dimensions of risk, encompassing genetic susceptibility, immune responses to bacillus exposure, social determinants, and epidemiological characteristics.^9^ ^,^ ^10^ However, these risk dimensions are often examined in isolation, resulting in a fragmented understanding of the factors that influence susceptibility and resistance to leprosy among contacts.

The present work seeks to provide a comprehensive analysis of risk factors associated with anti-PGL-I seropositivity and leprosy development among contacts in a highly endemic region. By integrating genetic, socioeconomic, environmental, and epidemiological factors, this study aims to enhance the understanding of outcomes associated with *M. leprae* exposure and to contribute valuable insights into leprosy control strategies.

## SUBJECTS AND METHODS

A retrospective cohort study was conducted, involving contacts of leprosy patients in the Almenara microregion, Minas Gerais, Brazil, with a follow-up period from 1999 to 2018. The Almenara microregion encompasses sixteen municipalities with a history of leprosy hyperendemicity and elevated rates of new case detection, making it an epidemiologically significant area for leprosy control.^11^

**Fig. 1:**
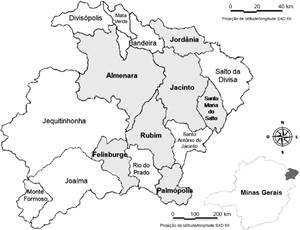
the microregion of Almenara, Minas Gerais, Brazil, with emphasis on the municipalities included in the study.

This study used data from three research projects led by the Centre for Studies and Research in Leprosy (NEPHANS) at the Federal University of Minas Gerais. These projects, conducted between 2011 and 2014 in seven hyperendemic municipalities within the Almenara microregion, specifically focused on Almenara, Felisburgo, Jacinto, Jordânia, Palmópolis, Rubim, and Santa Maria do Salto, as illustrated in Fig. 1.

*Study population* - The initial sample included index cases of leprosy and their contacts. The index case was defined as the first person in a household or on a shared property to develop leprosy. Contacts were individuals who lived in the same household, shared the same property, or had social relationships with an index case at the time of leprosy diagnosis or within the five years prior. “Joint property” referred to land with two or more houses on the same plot.

Index cases were identified from leprosy diagnosis records in the Notifiable Diseases Information System (SINAN) for patients diagnosed between January 1998 and July 2014 who were residents of the selected municipalities. Data were collected through home visits to index cases, conducted by trained nurses. During these visits, contacts were identified and recruited. Each contact completed a questionnaire covering sociodemographic, health, and epidemiological information. Biological samples were also collected, including 4 mL of whole blood in an EDTA tube for genetic polymorphism genotyping and a drop of blood for anti-PGL-I serology evaluation using the ML Flow rapid test. After collection, blood samples were placed in a thermal box and temporarily stored at -20ºC. The samples were then transported to the UFMG School of Nursing Laboratory for long-term storage at -80ºC.

Contacts underwent a clinical dermato-neurological evaluation to investigate signs of leprosy, and the physical examination included an assessment for the presence of any Bacille Calmette-Guérin (BCG) vaccine scar. This study included only contacts who lived in the same household or shared property with an index case and who consented to blood collection. Following the clinical dermato-neurological evaluation, individuals showing signs suggestive of leprosy but without a confirmed diagnosis by health services were excluded from the study.

*Variables studied* - The outcomes assessed in this study were anti-PGL-I seropositivity and the development of leprosy among contacts. Anti-PGL-I seropositivity was determined using the ML Flow rapid lateral flow test for *M. leprae*, which detects antibody reactivity to the antigenic glycolipid PGL-I. This test device includes a nitrocellulose strip with a detection reagent containing an anti-human IgM antibody and an absorption strip. The test was conducted following manufacturer’s instructions, which involved adding one drop of whole blood to the device’s receptacle, followed by five drops of buffer solution. After a five-minute incubation period, the results were interpreted based on the visibility of control and test lines.^12^

Leprosy development in contacts was evaluated through cross-validation using self-reported histories collected during interviews and by verifying leprosy notification records in the consolidated annual files of SINAN from 1999 to 2018. In Brazil, leprosy cases are diagnosed by medical professionals in both public or private health services, and are required to be reported to epidemiological surveillance agencies. Clinical and epidemiological data are documented on investigation forms and registered in SINAN. After processing the SINAN database, our search was conducted, restricting it to new cases reported in the municipalities of Minas Gerais, Brazil, during the specified period. Filters were applied to exclude relapsed cases, patient transfers, and misdiagnoses. To accurately identify individuals with common names, their age, date of birth, municipality of residence, and mother’s name were cross-referenced.

Risk factors were organised into three primary categories: sociodemographic determinants, genetic susceptibility, and exposure characteristics related to *M. leprae*. Sociodemographic determinants included gender, age, skin colour, educational level, and housing conditions, such as persons-per-room and average persons-per-bedroom. Persons-per-room was calculated by dividing the household population by the number of rooms, using the average number for the state of Minas Gerais, Brazil, as the reference standard. The average number of persons-per-bedroom was determined by dividing the household population by the number of bedrooms, with a standard of a maximum of two persons per bedroom, as recommended by the Brazilian Institute of Geography and Statistics (IBGE). Supplementary data (Table I) provides details on pre-existing variables in the original database as well as newly generated data for this study.

Exposure to *M. leprae* was assessed through the nature of contact (household contact, joint property, or social contact), the number of leprosy patients contacted, duration of close association with the patient until diagnosis, and clinical characteristics of the index case. “Joint property” refers to contacts who lived on a property with multiple households, including that of a leprosy patient. Clinical characteristics of the index case included the leprosy classification, bacilloscopic index (BI), and disability grade, which were recorded at the time of diagnosis using the WHO grading system (categorised as absent, grade 1, or grade 2). For analysis, BI results were categorised as positive or negative. These clinical data were extracted from SINAN archives. The number of BCG scars was also documented through physical examination.

*DNA extraction and genotyping* - Genetic susceptibility was assessed based on consanguinity with a leprosy patient and the presence of genetic polymorphisms in key genes associated with the immune response to *M. leprae*. Blood relatives within the third degree of consanguinity were considered. The selected single nucleotide polymorphisms (SNPs) included Toll-like receptor 1 (*TLR1*), rs4833095 and rs5743618; Nucleotide-binding oligomerisation domain protein 2 (*NOD2*), rs8057341, rs2066843, and rs751271; Leukotriene A4 Hydrolase (*LTA4H*), rs1978331 and rs17525495; Interferon-gamma (*IFNG*), rs2430561; and Interleukin 10 (*IL10*), rs1800871. These SNPs were chosen based on association studies conducted in the Brazilian population.^10^ ^,^ ^13^ ^,^ ^14^ ^,^ ^15^

Genomic DNA was extracted from frozen blood samples using the classic salting-out method^16^ and the Qiagen Flexigene Kit (catalog #51206; protocol available at: dx.doi.org/10.17504/protocols.io.se4ebgw). Genotyping was performed by amplifying DNA fragments through real-time polymerase chain reaction (qPCR). A genotyping assay with two primers and two probes specific to the target polymorphism was used to detect the presence or absence of allelic variants, indicated by changes in fluorescence signals of the amplified sequence. Details of the genotyping assays are provided in Supplementary data (Table II). The reaction volume was set to 20 µL, consisting of 20 ng of genomic DNA and a 1X reaction buffer containing master mix and genotyping assay reagents. RT-PCR reactions were conducted on step one real-time PCR systems (protocol available at: dx.doi.org/10.17504/protocols.io.sfzebp6).

*Statistical analysis* - After integrating the databases and removing duplicate entries, the chi-square test (χ²) was applied to assess the agreement of genetic variants, using the hardy-Weinberg equilibrium (HWE). For both bivariate and multivariate analyses, binary logistic regression was performed using the generalised estimating equations (GEE) method. Since contacts were clustered within households, individual entries could not be treated as independent events. Therefore, the household was designated as the observation cluster. The GEE method accounts for the correlation structure of household characteristics, estimates the residual similarity within clusters, and uses this estimated correlation to re-estimate regression parameters and calculate standard errors. This approach provides more efficient estimates than conventional methods when handling correlated data structures.^17^

Multivariate analyses were conducted using a backward selection method, with a p-value threshold of < 0.20 to include covariates in the models. Due to the large number of explanatory variables, separate multivariate analyses were performed for each dimension. The final model retained only explanatory variables that showed statistical significance within each dimension. The measure of association was the odds ratio (OR), provided with a 95% confidence interval (95% CI). Statistical significance was set at a 5% level (p < 0.05). All statistical analyses were conducted using Stata, version 12.0.

*Ethical aspects* - The studies involving human participants were reviewed and approved by the Research Ethics Committee of the Federal University of Minas Gerais, Brazil, and conducted in accordance with the Declaration of Helsinki (1975), as revised in 1983. The Research Ethics Committee granted approval for the projects under the following protocols: ETIC no. 0454.0.203.000-10 (2010), no. 158/09 (2009), and CAAE: 01910312.5.0000.5149 (2012). All participants were invited to participate voluntarily and provided written informed consent. For minors, written informed consent was obtained from their parents or legal guardians.

All relevant data are included within the paper and its Supplementary data. The authors will make the raw data supporting the conclusions of this article available without undue restriction.

## RESULTS

**Fig. 2:**
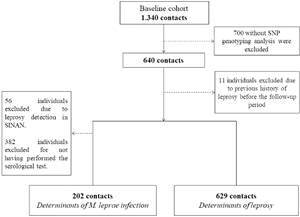
flowchart to select the contact sample for each outcome

The cohort consisted of 629 contacts, with females representing 54.2% (341/629) and a median age of 37 years. Approximately 80% (502/629) of the contacts were household members or shared joint property with the leprosy case, while 20% (127/629) were social contacts. Supplementary data (Table III) provides detailed characteristics of the contacts included in this study. Fig. 2 presents a flowchart of the sample selection process at each study stage, including reasons for participant exclusion.

Anti-PGL-I serology was performed on 205 contacts, of whom 35 tested positive. Three contacts who developed leprosy were excluded from this analysis, resulting in a final sample size of 202 contacts, among whom 33 (16.3%) showed antibody reactivity. Black and brown-skinned contacts had a significantly higher likelihood of seropositivity when compared to white contacts, with non-white contacts nearly five times more likely to be seropositive for the PGL-I antigen (OR = 4.77; 95% CI: 1.04-21.9; p = 0.044). Additionally, living with a MB index case increased the likelihood of positive serology (OR = 5.48; 95% CI: 2.04-14.7; p = 0.001), as did contact with patients exhibiting grade 1 (OR = 2.93; 95% CI: 1.21-7.10; p = 0.018) or grade 2 disabilities upon diagnosis (OR = 8.99; 95% CI: 2.58-31.3; p = 0.001). Table I presents the results of the bivariate analysis of factors associated with anti-PGL-I seropositivity.

**TABLE I:** Sociodemographic determinants, genetic susceptibility, and characteristics of exposure to *Mycobacterium leprae* associated with anti-phenolic glycolipid-I (anti-PGL-I) seropositivity in contacts

Variables	Anti-PGL-I serology		*χ2* (p-value)	OR (95%CI)	p-value
n (%)	
Positive	Negative
Sociodemographic determinants					
Sex					
Female	20 (60.6)	92 (54.4)	0.425 (0.514)	1.24 (0.58-2.66)	0.573
Male	13 (39.4)	77 (45.6)		1	
Age (years)					
< 15	10 (30.3)	41 (24.3)	1.187 (0.756)	1.75 (0.49-6.28)	0.388
15-29	9 (27.3)	39 (23.1)		1.65 (0.45-6.03)	0.448
30-59	10 (30.3)	62 (36.7)		1.19 (0.34-4.16)	0.789
≥ 60	4 (12.1)	27 (16.0)		1	-
Skin colour					
White	2 (6.5)	39 (23.8)	4.715 **(0.030)**	1	-
Brown/Black	29 (93.5)	125 (76.2)		4.77 (1.04-21.9)	0.044
Level of education^*^					
Low	27(81.8)	133(78.7)	4.2890 (0.117)	0.20 (0.03-1.48)	0.114
Medium	4 (12.1)	34 (20.1)		0.12 (0.01-1.10)	0.061
High	2 (6.1)	2 (1.2)		1	-
Persons-per-room in the household					
≤ 0.6	12 (36.4)	73 (43.2)	0.529 (0.467)	1	-
> 0.6	21 (63.6)	96 (56.8)		1.33 (0.60-2.95)	0.488
Average number of persons-per-bedroom					
≤ 2	26 (78.8)	132 (78.1)	0.008 (0.931)	1	-
> 2	7 (21.2)	37 (21.9)		0.94 (0.36-2.46)	0.900
Genetic susceptibility					
Consanguinity with the index case					
No	9 (27.3)	57 (33.7)	0.523 (0.470)	1	-
Yes	24 (72.7)	112 (66.3)		1.38 (0.60-3.17)	0.445
**Characteristics of exposure to *M. leprae***					
Contact characteristics					
Household	30 (90.9)	157 (92.9)	0.159 (0.690)	1	-
Joint property or social contact	3 (9.1)	12 (7.1)		1.49 (0.51-4.35)	0.464
Number of leprosy patients in the household/house on the same lot					
1	25 (75.7)	138 (81.7)	0.617 (0.432)	1	-
> 1	8 (24.3)	31 (18.3)		1.45 (0.58-3.63)	0.433
Length of time of close association					
≤ 5 years	8 (24.2)	27 (16.0)	1.317 (0.251)	1	-
> 5 years	25 (75.8)	142 (84.0)		0.60 (0.24-1.50)	0.277
Leprosy classification					
Paucibacillary	5 (15.1)	83 (49.4)	13.148 **(< 0.0001)**	1	-
Multibacillary	28 (84.9)	85 (50.6)		5.48 (2.04-14.7)	0.001
Bacilloscopic index					
Negative	16 (59.3)	75 (65.2)	0.337 (0.561)	1	-
Positive	11 (40.7)	40 (34.8)		1.30 (0.53-3.17)	0.563
Disability grade					
Absent	8 (26.7)	93 (56.7)	13.818 **(0.001)**	1	-
Grade 1	16 (53.3)	63 (38.4)		2.93 (1.21-7.10)	0.018
Grade 2	6 (20.0)	8 (4.9)		8.99 (2.58-31.3)	0.001
BCG scar					
Absent	6 (18.2)	33 (19.6)	0.917 (0.632)	1	-
1 scar	14 (42.4)	83 (49.4)		0.96 (0.34-2.73)	0.933
2 scars	13 (39.4)	52 (31.0)		1.47 (0.50-4.27)	0.483

Values in bold represent significant results. *Low: less than primary, primary and lower secondary education; medium: upper secondary education and post-secondary; high: tertiary education; OR: odd ratio.

No association was observed between consanguinity with the index case and seropositivity; thus, only sociodemographic determinants and characteristics of *M. leprae* exposure were included in the multivariate analysis. The final model identified skin colour, leprosy classification, and disability grade as significant variables (Table II). Living with an index case presenting MB leprosy and grade 2 disabilities significantly increased the chances of anti-PGL-I seropositivity among contacts (OR = 3.01; 95% CI: 1.02-8.86; p = 0.045 and OR = 4.43; 95% CI: 1.08-18.1; p = 0.038, respectively).

**TABLE II:** Multivariate analysis of risk factors for anti-phenolic glycolipid-I (anti-PGL-I) seropositivity in contacts

Variable	OR	95%CI	p-value
Skin colour			
White	1	-	-
Brown/Black	3.61	0.81-16.1	0.093
Leprosy classification			
Paucibacillary	1	-	-
Multibacillary	3.01	1.02-8.86	0.045
Disability grade			
Absent	1	-	-
Grade 1	2.15	0.82-5.64	0.119
Grade 2	4.43	1.08-18.1	0.038

Values in bold represent significant results. OR: odd ratio.

In this cohort, 56 cases of leprosy were detected, with 38% (21/56) of the cases occurring within the first year after the index case’s diagnosis, and 82% (46/56) occurring after the first year. Among the contacts who developed leprosy, 54% were classified as paucibacillary (PB) and 46% as MB. In the bivariate analysis, factors like age, contact characteristics, and the number of patients in the contact environment were associated with leprosy (Table III). Leprosy was nearly five times more likely to be detected in contacts aged 15 to 29 years (OR = 4.78; 95% CI: 1.76-12.98; p = 0.003). Analysis of the type of contact revealed that contacts living on joint property were three times more likely to develop leprosy (OR = 3.35; 95% CI: 1.42-7.92; p = 0.014), while social contacts had a lower chance of developing the disease (OR = 0.28; 95% CI: 0.10-0.83; p = 0.032). Sharing a household or property with more than one patient showed a strong association with leprosy detection; however, this variable was excluded from the multivariate analysis due to limited variability within the affected group. Notably, 98% of the contacts who developed leprosy had lived with more than one patient.

**TABLE III:** Sociodemographic determinants and characteristics of exposure to *Mycobacterium leprae* associated with the development of leprosy in contacts

Variables	Leprosy		*χ2* (p-value)	OR (95%CI)	p-value
	n (%)				
	Affected	Healthy			
Sociodemographic determinants					
Sex					
Female	29 (51.8)	312 (54.5)	0.146 (0.702)	0.89 (0.51-1.54)	0.675
Male	27 (48.2)	261(45.5)		1	-
Age (years)					
< 15	5 (8.9)	105 (18.3)	14.579 **(0.002)**	1.17 (0.34-4.07)	0.802
15-29	22 (39.3)	117 (20.4)		4.44 (1.64-12.01)	0.003
30-59	24 (42.9)	231 (40.3)		2.40 (0.90-6.39)	0.079
≥ 60	5 (8.9)	120 (20.9)		1	-
Skin colour					
White	9 (16.4)	106 (20.0)	0.417 (0.518)	1	-
Brown/Black	46 (83.6)	424 (80.0)		1.26 (0.60-2.65)	0.537
Level of education^*^					
Low	36 (73.5)	461 (81.3)	3.5562 **(0.169)**	0.57 (0.29-1.11)	0.099
Medium	13 (26.5)	96 (16.94)		1	-
High	0 (0)	7 (1.8)		-	-
Persons-per-room in the household					
≤ 0.6	24 (42.9)	249 (43.5)	0.007 (0.931)	1	-
> 0.6	32 (57.1)	324 (56.5)		1.04 (0.58-1.86)	0.890
Average number of persons-per-bedroom					
≤ 2	45 (80.4)	454 (79.2)	0.039 (0.843)	1	-
> 2	11 (19.6)	119 (20.8)		0.94 (0.46-1.94)	0.868
**Characteristics of exposure to *M. leprae***					
Contact characteristics					
Household	44 (78.6)	422 (73.6)	13.22**(0.001)**	1	-
Joint property	8 (14.3)	28 (4.9)		2.87 (1.24-6.67)	0.014
Social contact	4 (7.1)	123 (21.5)		0.31 (0.10-0.90)	0.032
Number of leprosy patients in the household/house on the same lot					
1	1 (1.9)	443 (77.6)	135.39 **(< 0.0001)**	1	-
> 1	52 (98.1)	128 (22.4)		178.8 (24.9-1285.4)	< 0.0001
Length of time of close association					
≤ 5 years	1 (25)	208 (36.3)	0.220 (0.639)	1	-
> 5 years	3 (75)	365 (63.7)		1.75 (0.18-16.9)	0.627
Leprosy classification					
Paucibacillary	17 (33.3)	221 (39.8)	0.824 (0.364)	1	-
Multibacillary	34 (66.7)	334 (60.2)		1.35 (0.71-2.55)	0.357
Bacilloscopic index					
Negative	25 (73.5)	318 (70.8)	0.112 (0.737)	1	-
Positive	9 (26.5)	131 (29.2)		0.94 (0.41-2.16)	0.879
Disability grade					
Absent	24 (47.1)	294 (55.5)	1.491 (0.474)	1	-
Grade 1	23 (45.1)	207 (39.0)		1.42 (0.76-2.68)	0.273
Grade 2	4 (7.8)	29 (5.5)		1.75 (0.54-5.60)	0.347
BCG scar					
Absent	19 (33.9)	159 (27.8)	1.166 (0.558)	1	-
1	24 (42.9)	284 (49.7)		0.72 (0.38-1.37)	0.320
2	13 (23.2)	129 (22.6)		0.87 (0.41-1.83)	0.711

Values in bold represent significant results. *Low: less than primary, primary and lower secondary education; medium: upper secondary education and post-secondary; high: tertiary education; OR: odd ratio.

The genotypic distribution of SNPs, their alignment with HWE, and the association between genetic characteristics and leprosy susceptibility are detailed in Supplementary data (Table IV). All SNPs were consistent with HWE. Notably, the rs2066843 polymorphism in the NOD2 gene was associated with an increased susceptibility to leprosy. Contacts carrying the heterozygous CT genotype for the NOD2 SNP had a higher likelihood of developing leprosy when compared to those with the wild-type CC allele (OR = 1.93; 95% CI: 1.01-3.68; p = 0.047). This association remained statistically significant after adjusting for sex and age (OR = 1.95; 95% CI: 1.05-3.75; p = 0.045).

The final model for leprosy included contact characteristics, consanguinity with the index case, and the SNP rs2066843 in the NOD2 gene. In the bivariate analysis, consanguinity with the index case approached statistical significance (p = 0.05), and was therefore included in the multivariate analysis. Both having a blood relationship with the index case and being a household contact showed significant associations with leprosy detection among contacts (Table IV). Social contacts had a lower chance of leprosy detection when compared to household contacts (OR = 0.27; 95% CI: 0.09-0.78; p = 0.016). Sharing the same property was identified as a risk factor for leprosy (OR = 2.84; 95% CI: 1.21-6.67; p = 0.017).

**TABLE IV:** Multivariate analysis of risk factors associated with the development of leprosy in contacts

Variable	OR	95%CI	p-value
Contact characteristics			
Household	1	-	-
Joint property	2.84	1.21-6.67	0.017
Social contact	0.27	0.09-0.78	0.016
Consanguinity with the index case			
No	1	-	-
Yes	2.56	1.18-5.54	0.017
*NOD2_SNP rs2066843* ^*a*^			
*CC* genotype	1	-	-
CT/TT	1.76	0.88-3.53	0.112
CC	1	-	-
CT	1.89	0.93-3.85	0.077
TT	0.70	0.07-6.55	0.758

*a*: analysis adjusted for sex and age; OR: odd ratio. Values in bold represent significant results.

## DISCUSSION

In this study, we found that living with a MB patient, especially those with physical disabilities upon diagnosis, was associated with *M. leprae* infection. Patients with positive bacilloscopy and MB clinical forms are a significant source of *M. leprae* transmission in endemic regions.^5^ ^,^ ^18^ ^,^ ^19^ One study, involving untreated leprosy patients, identified *M. leprae* DNA in the nasal and oral mucosa of 56% and 44% of lepromatous leprosy patients, respectively.^20^ Moreover, research in countries with varying endemicity levels has shown that anti-PGL-1 seropositivity is commonly observed among contacts of MB cases with positive dermal smear microscopy.^7^ ^,^ ^20^ ^,^ ^21^

In our study, the variable most strongly associated with leprosy in contacts was the number of cases in the living environment. Association studies have consistently shown a link between leprosy development and the clinical characteristics of the index case.^5^ ^,^ ^6^ ^,^ ^9^ ^,^ ^19^ ^,^ ^22^ ^,^ ^23^ While contacts of MB patients and those with physical disabilities face increased infection risk, the progression from infection to clinical manifestations depends not only on exposure to the bacillus, but also on the intensity of that exposure.

In 2021, 8.14% of newly diagnosed leprosy cases in Brazil exhibited grade 2 disabilities,^4^ indicating that active case finding and contact surveillance have not received adequate emphasis in Brazil’s leprosy control efforts. A study conducted in India revealed that leprosy patients with grade 2 disabilities experienced an average diagnostic delay of 23.2 months. This delay underscores both a lack of community awareness about leprosy and operational challenges in early detection within healthcare services.^24^ These findings highlight the critical role of health services, particularly primary healthcare, in achieving the World Health Organization’s objectives of interrupting transmission and preventing disease.^1^

Consanguinity with the index case was associated with a higher likelihood of leprosy detection among contacts, consistent with prior studies indicating up to a threefold increased risk for consanguineous contacts as compared to non-consanguineous ones.^6^ ^,^ ^9^ The progression from exposure to infection and disease is influenced by host genetics in innate and adaptive immunity. However, a purely biological model cannot fully explain the complexity of the leprosy phenotype.

In our analysis of genetic variants associated with leprosy, we found that individuals carrying the heterozygous genotype for the rs2066843 SNP in the NOD2 gene had a higher risk of developing leprosy. Previous studies have reported associations between NOD2 polymorphisms and leprosy susceptibility, highlighting the potential role of this gene in future research on leprosy susceptibility among contacts.^10^ ^,^ ^14^ Studies have shown that NOD2-deficient mice stimulated with mycobacterial antigens exhibit reduced TNF-α and IL-12 production by macrophages and diminished T-cell recruitment. Upon infection, these mice demonstrated a lower Th1 cytokine response (TNF-α and IFN-γ) and reduced CD4+ and CD8+ T cell production.^25^ The absence of functional evidence on NOD2’s role, combined with the inconsistent genetic association findings across different populations, may result from the polygenic inheritance patterns characterising leprosy, warranting further studies to explore this area.^26^ Other genetic polymorphisms in the genes evaluated in this study were not associated with leprosy in contacts. Given that leprosy is a polygenic disease, these findings support the potential use of consanguinity as a proxy for genetic susceptibility.

This study found that both household contacts and those living on joint properties were at a higher risk for leprosy. Contacts residing on joint properties share more living spaces with patients and are exposed to similar sociodemographic factors within family units, such as poorer housing conditions. Leprosy development in these contacts appears to be associated with increased *M. leprae* transmission facilitated by household density. Analysis of living conditions among contacts revealed a significantly higher average number of persons per bedroom for those living on the same property as the index case (χ² = 12.51; p = 0.002). An initial analysis of infection determinants also showed that brown and black contacts showed a higher risk of infection when compared to white contacts. Most non-white contacts resided in overcrowded households, underscoring the impact of social determinants on infection and leprosy development.^5^

The effect of social inequality on disease control is substantial. According to the United Nations Human Rights Council, social inequality poses a significant barrier to Brazil’s efforts to achieve leprosy-free status, as poverty limits the effectiveness of disease control measures implemented by health services.^27^ Another factor increasing the risk of leprosy in household contacts may be indirect exposure to *M. leprae* through environmental sources. Studies have identified viable bacilli in soil and water from households with leprosy cases, emphasising the role of indirect environmental contamination.^28^ ^,^ ^29^

The presence of a BCG vaccination scar was not associated with the evaluated outcomes, despite evidence from previous studies indicating a protective effect of BCG immunisation among contacts.^6^ ^,^ ^19^ In Brazil, leprosy control programs recommend BCG vaccination for asymptomatic contacts. Among the 180 contacts of multiple leprosy patients, 27% (n = 48) lacked a BCG vaccination scar, highlighting weaknesses in health services’ monitoring and provision of immunoprophylaxis for contacts. The results of the bivariate analysis indicated a higher likelihood of leprosy among contacts aged 15 to 29 years. The increased incidence of leprosy in young individuals may reflect early exposure to *M. leprae* in this region, possibly due to the ongoing transmission of the bacillus.

It is important to acknowledge limitations in the cohort follow-up period of this analysis, as some healthy participants may still develop the disease. Additionally, the regression model may be influenced by infection risk factors. Anti-PGL-I serology has limited sensitivity in predicting leprosy development, particularly in PB cases and contacts who have not developed significant antibody levels.^7^ ^,^ ^22^ ^,^ ^30^ ^,^ ^31^ Thus, relying solely on serology for contact surveillance has limitations.

Case definitions in this study relied on SINAN notifications. The SINAN database is subject to underreporting, given health services’ variable operational capacity to detect and diagnose leprosy. Therefore, categorising cases based on SINAN records is a limitation. Future studies could address this by directly following healthy and infected contacts to reduce risks of underreporting.

A limitation in the genetic analysis is that the sample of contacts included blood relatives, potentially biasing allele frequency analysis and affecting genetic outcomes. As this study is an exploratory pilot with convenience sampling, future research should build on these findings by including more genetic variables and a larger sample. It is necessary to accurately assess the importance of identified variables, to identify the penetrance of genetic variants, and to ensure the reproducibility of results. Additionally, future studies could incorporate more sensitive tests to detect subclinical infections, such as the evaluation of bacillary DNA in nasal mucosa, skin smears, and blood.

The findings of this study underscore the importance of protecting contacts from *M. leprae* infection, as progression to clinical leprosy is influenced by individual genetic susceptibility. The high infectivity yet low pathogenicity of the leprosy bacillus highlights the need to prioritise infection control in endemic areas. In such regions, subclinical infections are common, and infected contacts may perpetuate transmission.^7^ ^,^ ^23^ Consequently, only diagnosing and treating symptomatic patients is not enough to eliminate leprosy in areas with high epidemiological significance.

To enhance contact surveillance and identify individuals with subclinical infection who may benefit from preventive chemoprophylaxis and closer health service monitoring, strategies, such as the Mitsuda test, serological tests, and genetic assessments of bacillus presence, as well as viability in biological samples can be used together with dermato-neurological exams and BCG immunisation.^22^ Our analysis of *M. leprae* infection and leprosy detection among contacts also reveals that the risk of these outcomes is associated with modifiable social determinants and challenges in accessing and operationalising health services.

The models analysed in this study have identified patterns indicating risk factors for infection and leprosy among contacts. Social vulnerability is a precursor to these outcomes, as bacillus transmission and infection are fostered by unfavourable social conditions and individual susceptibility. Strengthening leprosy control programs and ensuring universal, equitable access to healthcare and social policies for affected individuals and their contacts are imperative.

Effective contact surveillance is essential to enhancing leprosy control efforts. Passive detection and multidrug therapy are inadequate to interrupt transmission. Surveillance requires examining household contacts, neighbours, and social contacts, actively searching for new cases, providing chemoprophylaxis with rifampin, and administering BCG vaccination. People affected by leprosy should have access to free healthcare, adequate food, housing, basic sanitation, and education. Ensuring the right to social and political participation without discrimination is also crucial. These measures should aim to reduce poverty and improve the quality of life for affected communities.
