# Supplementary material for: Anti-PGL-I seropositivity and development of leprosy in contacts: a comprehensive analysis of sociodemographic determinants, genetic susceptibility, and exposure characteristics to Mycobacterium leprae
Source: Mem Inst Oswaldo Cruz. 2025 May 26;120:e240061. doi: 10.1590/0074-02760240061 (PMC12113343; doi:10.1590/0074-02760240061)
Supplement: Supplementary file 1 [file 1678-8060-mioc-120-e240061-s.pdf]

TABLE I

Source of variables

Original database	Created/Analysed for this study (New data)
Sex	Categorised age
Age (years)	Persons-per-room in the household
Skin colour	Average number of persons-per-bedroom
Education	TLR1_ rs5743618
Residence area	NOD2_ rs8057341
Number of persons in the household	*NOD2_rs206684*3
Number of rooms in the household	NOD2_ rs751271
Number of bedrooms in the household	LTA4H_ rs1978331
Consanguinity with the index case	LTA4H_ rs17525495
Leprosy classification[a]	IFNG_ rs2430561
Bacilloscopic index[a]	IL10_ rs1800871
Disability grade[a]	Length of time of close association (categorised)
BCG scar	Contact characteristics
	Number of leprosy patients in the household/joint property

a: clinical data obtained from the SINAN archives.

TABLE II

TaqMan® genotyping assays from Applied Biosystems | Thermo Fisher Scientific, used for real-time polymerase chain reaction (PCR) allelic discrimination

Gene_SNP	Assay ID
TLR1_ rs4833095	C__44103606_ 10
TLR1_ rs5743618	C_175679112_10
NOD2_ rs8057341	C___3017466_10
NOD2_rs2066843	C__11717469_10
NOD2_ rs751271	C___1384442_10
LTA4H_ rs1978331	C__11700137_1_
LTA4H_ rs17525495	C__25593629_10
IFNG_ rs2430561	AH20TEB
IL10_ rs1800871	C___1747362_10

cc BY

TABLE III

General characteristics of the contacts

Variables	n	%
Sex		
Female	341	54.2
Male	288	45.8
Age (years)	Median (Minimum-Maximum)	37 (2 - 99)
Categorised age		
<15	110	17.5
15-29	139	22.1
30-59	255	40.5
≥ 60	125	19.9
Skin colour		
White	115	19.7
Black	46	7.9
Brown	424	72.5
Level of education*		
Low	135	21.9
Medium	362	58.8
High	119	19.3
Residence area		
Rural	46	7.3
Urban	583	92.7
Number of persons in the household	Median (Minimum-Maximum)	4 (1 - 10)
Number of rooms in the household		6 (2 - 17)
Number of bedrooms in the household		3 (0 - 6)
Persons-per-room in the household		
≤ 0.6	273	43.4
> 0.6	356	56.6
Average number of persons-per-bedroom		
≤ 2	499	79.3
2	130	20.7
Consanguinity with the index case		
No	169	27
Yes	458	73
Length of time of close association (years)	Median (Minimum-Maximum)	11 (< 1 - 74)
Length of time of close association (categorised)		
< 5	209	36.2
5	368	63.8
Contact characteristics		
Household	466	74.1
Joint property	36	5.7
Social contact	127	20.2
Number of leprosy patients in the household/joint property		
1	444	71.2
> 1	180	28.8

↳

Variables	n	%
Leprosy classification		
Paucibacillary	238	39.3
Multibacillary	368	60.7
Bacilloscopic index		
Negative	343	71.0
Positive	140	29.0
Disability grade		
Absent	318	54.7
Grade 1	230	39.6
Grade 2	33	5.7
BCG scar		
Absent	178	28.3
1 scar	308	49.0
2 scars	142	22.6

*Low: less than primary, primary and lower secondary education; medium: upper secondary education and post-secondary; high: tertiary education.

TABLE IV

Genetic characteristics associated with leprosy in contacts

Variables	Leprosy n (%) Affected	Healthy	HWE[a] $\chi2$ (p-value)	OR[b] (95%CI)	p-value	OR[bc] (95%CI)	p-value
Consanguinity with the index case							
No	8 (14.8)	161 (28.1)	-	1	-	NA[†]	-
Yes	46 (85.2)	412 (71.9)		2.13 (1.0-4.55)	0.050		-
TLR1_SNP rs4833095							
C	55 (52.9)	545 (48.3)		1	-	-	-
T	49 (47.1)	583 (51.7)		0.83 (0.56-1.25)	0.373		
CC	14 (26.9)	124 (23.9)		1	-	1	-
CT/TT	38 (73.1)	429 (76.1)		0.88 (0.46-1.67)	0.689	0.86 (0.45-1.64)	0.648
CC	14 (26.9)	135 (23.9)	0.068 (0.967)	1	-	1	-
CT	27 (51.9)	275 (48.8)		0.99 (0.50-1.94)	0.969	1.04 (0.48-2.25)	0.923
TT	11 (21.2)	154 (27.3)		0.68 (0.30-1.57)	0.372	0.59 (0.21-1.61)	0.301
Total	52 (8.44)	564 (91.6)					
TLR1_SNP rs5743618							
T	54 (65.9)	458 (62.4)		1	-	-	-
G	28 (34.1)	276 (37.6)		0.86 (0.53-1.39)	0.540		
TT	17(41.5)	148 (40.3)		1	-	1	-
TG/GG	24 (58.5)	219 (59.7)		0.99 (0.52-1.88)	0.971	0.91 (0.48-1.74)	0.790
TT	17 (41.5)	148 (40.3)	0.640 (0.726)	1	-	1	-
TG	20 (48.8)	162 (44.1)		1.10 (0.56-2.16)	0.772	1.04 (0.53-2.04)	0.901
GG	4 (9.8)	57 (15.5)		0.64 (0.21-1.93)	0.431	0.56 (0.19-1.69)	0.305
Total	41 (10.1)	367 (89.9)					
NOD2_SNP rs8057341							
G	78 (70.9)	776 (69.0)		1	-	-	-
A	32 (29.1)	348 (31.0)		0.97 (0.59-1.41)	0.685		
GG	27 (49.1)	276 (49.1)		1	-	1	-
GA/AA	28 (50.9)	286 (50.9)		0.98 (0.56-1.71)	0.950	1.02 (0.58-1.77)	0.952
GG	27 (49.1)	276 (49.1)	2.500 (0.287)	1	-	1	-
GA	24 (43.6)	224 (39.9)		1.07 (0.60-1.91)	0.809	1.10 (0.61-1.96)	0.750
AA	4 (7.3)	62 (11.0)		0.64 (0.22-1.91)	0.428	0.70 (0.24-2.07)	0.519
Total	55 (8.9)	562 (91.1)					
NOD2_SNP rs2066843							
C	63 (75.0)	616 (82.6)		1	-	-	-
T	21 (25.0)	130 (17.4)		1.58 (0.93-2.68)	0.090		
CC	22 (52.4)	253 (67.8)		1	-	1	-
CT/TT	20 (47.6)	120 (32.2)		1.85 (0.98-3.50)	0.058	1.82 (0.96-3.46)	0.067
CC	22 (52.4)	253 (67.8)	1.032 (0.597)	1	-	1	-
CT	19 (45.2)	110 (29.5)		1.93 (1.01-3.68)	**0.047**	1.95 (1.02-3.75)	**0.045**
TT	1 (2.4)	10 (2.7)		1.04 (0.12-8.98)	0.972	0.80 (0.09-6.77)	0.838
Total	42 (10.1)	373 (89.9)					

Variables	Leprosy n (%)		HWE[a] $\chi 2$ (p-value)	OR[b] (95%CI)	p-value	OR[bc] (95%CI)	p-value
	Affected	Healthy					
NOD2_SNP rs751271							
G	56 (66.7)	444 (59.8)		1	-	-	-
T	28 (33.3)	298 (40.2)		0.75 (0.46-1.20)	0.226		
GG	17 (40.5)	144 (38.8)		1	-	1	-
GT/TT	25 (59.5)	227 (61.2)		0.92 (0.49-1.75)	0.808	0.99 (0.52-1.89)	0.991
GG	17 (40.5)	144 (38.8)	4.535 (0.104)	1	-	1	-
GT	22 (52.4)	156 (42.0)		1.18 (0.61-2.29)	0.614	1.31 (0.67-2.55)	0.430
TT	3 (7.1)	71 (19.1)		0.36 (0.10-1.23)	0.104	0.37 (0.11-1.27)	0.114
Total	42 (10.2)	371 (89.8)					
LTA4H_SNP rs1978331							
G	75 (89.3)	678 (90.6)		1	-	-	-
A	9 (10.7)	70 (9.4)		1.16 (0.56-2.42)	0.688		
GG	34 (81.0)	308 (82.4)		1	-	1	-
GA/AA	8 (19.0)	66 (17.6)		1.10 (0.49-2.49)	0.809	1.08 (0.48-2.44)	0.855
GG	34 (81.0)	308 (82.4)	0.348 (0.840)	1	-	1	-
GA	7 (16.7)	62 (16.6)		1.03 (0.44-2.41)	0.950	1.01 (0.43-2.38)	0.977
AA	1 (2.4)	4 (1.1)		2.39 (0.28-20.6)	0.427	2.04 (0.24-17.2)	0.514
Total	42 (10.1)	374 (89.9)					
LTA4H_SNP rs17525495							
A	38 (46.3)	382 (51.9)		1	-	-	-
G	44 (53.7)	354 (48.1)		1.25 (0.79-1.97)	0.340		
AA	10 (24.4)	102 (27.7)		1	-	1	-
AG/GG	31 (75.6)	266 (72.3)		1.16 (0.56-2.42)	0.685	1.06 (0.51-2.20)	0.886
AA	10 (24.4)	102 (27.7)	0.840 (0.657)	1	-	1	-
AG	18 (43.9)	178 (48.4)		1.01 (0.46-2.25)	0.970	0.94 (0.43-2.08)	0.882
GG	13 (31.7)	88 (23.9)		1.48 (0.62-3.50)	0.375	1.28 (0.54-3.06)	0.572
Total	41 (10.0)	368 (90.0)					
IFNG_SNP rs2430561							
A	59 (70.2)	457 (61.9)		1	-		
T	25 (29.8)	281 (38.1)		0.69 (0.42-1.13)	0.137		
AA	21 (50.0)	133 (36.0)		1	-	1	-
AT/TT	21 (50.0)	236 (64.0)		0.56 (0.30-1.06)	0.077	0.58 (0.30-1.09)	0.090
AA	21 (50.0)	133 (36.0)	3.813 (0.149)	1	-	1	-
AT	17 (40.5)	191 (51.8)		0.55 (0.28-1.08)	0.084	0.57 (0.29-1.11)	0.100
TT	4 (9.5)	45 (12.5)		0.61 (0.21-1.77)	0.362	0.61 (0.21-1.79)	0.372
Total	42 (10.2)	369 (89.8)					
IL-10_SNP rs1800871							
G	49 (59.8)	463 (62.4)		1	-		
A	33 (40.2)	279 (37.6)		1.12 (0.70-1.78)	0.640		
GG	13 (31.7)	143 (38.5)		1	-	1	-
GA/AA	28 (68.3)	228 (61.5)		1.31 (0.67-2.59)	0.427	1.29 (0.66-2.53)	0.460
GG	13 (31.7)	143 (38.5)	0.324 (0.850)	1	-	1	-
GA	23 (56.1)	177 (47.7)		1.38 (0.69-2.78)	0.361	1.36 (0.68-2.72)	0.386
AA	5 (12.2)	51 (13.7)		1.06 (0.37-3.08)	0.912	1.03 (0.35-2.99)	0.958
Total	41 (10.0)	371 (90.0)					

a: Hardy-Weinberg Equilibrium; [†]not analysed; *b*: odds ratio was calculated by binary logistic regression using the generalised estimating equations method; *c*: analysis adjusted for sex and age. Values in bold represent significant results.
